# Editorial

**Published:** 1882-09

**Authors:** 


### EDITORIAL.

Our September, October and November Issues we propose to make small on account of press of business, but will make up the deficiency in the December number. We hope that some of our collaborators will send us some matter at once.

Bacteria.—We hope that all of our readers will peruse carefully the first article in this issue of the journal. Its author, F. Y. Clark, M. D., of N. Y., has for the past twenty-five years been an earnest investigator of this microscopical consumer of all organized matter. So far as we know, he is the original discoverer of bacteria of the teeth, and deserves great credit for opening this avenue for scientific investigation. In 1871 he read an essay on this subject before the Southern Dental Association, then in session at Charleston, S. C., which received considerable attention. It was published and criticised in several of the journals. Through invitation he read another essay in 1879 before the Connecticut Valley Dental Association, which was published in *Johnston's Dental Miscellany*. Last winter he brought the subject to the attention of the Microscopical Society of New York.

Professional Education.—The father of scientific dentistry, Prof. Chapin A. Harris, saw from the beginning of his useful career that the proper method to make dentistry a profession, was to make it a specialty of medicine. He therefore, in 1839, before founding the first dental school in the world, made an effort to establish a dental department in the School of Medicine of the Maryland University, when dentistry was, in a general sense, a trade, and the majority of those in the practice were barbers, blacksmiths, etc., who were totally unfit to enter the University, and for this reason his plans failed to be acceptable to the medical faculty. He abandoned them and applied to the State Legislature of Maryland for a charter for a dental college, which was obtained. From this first dental school a scaffold was erected, and the medical fraternity is no longer ashamed to acknowledge dentistry as a specialty of medicine. This fall, his old "ideal" school stands upon the identical spot and original plan which his noble mind conceived years ago, beginning its first annual session with more than fifty students, and it is the Dental Department of the University of Maryland.

Let all of these old scaffolds be torn down, in order that the beautiful building (now partly hidden) may show its towering head of science and professional dignity. Let the preceptor do his duty in selecting his students to send to the scientific schools. We also urge the colleges to be more careful in accepting educated gentlemen adapted to the profession of their choice, than making a long list of matriculants. Having secured suitable material, keep it within the college walls fully two years—devoted to study and practice. Pour into this cement lessons of science, charity, professional honor, cleanliness of person and character, both by "precept and example." If preceptor, professor and student will act their part well in each step of scientific education, we will have indeed a noble profession.

THE TRADE-MARK CONTROVERSY AGAIN.—Dr. Horatio R. Bigelow, in a letter to a publication purporting to be a medical journal issued in St. Louis, sarcastically endeavors to discount the effect of our editorial in the April number of the INDEPENDENT PRACTITIONER, entitled "A Chapter on Medical Ethics." In our article we desired to be perfectly just and fair. We resented in as decisive a manner as we were able, the inference to be drawn from Dr. Bigelow's article in the *N. E. Medical Monthly*. We refrained from direct personalities, and only characterized Dr. B.'s article in such terms as we still believe it deserved. As to Dr. Bigelow himself, we are entirely indifferent. His innocent attempts at sarcasm do no harm, and if they amuse him, why, let him indulge himself to his heart's content.

IN OUR AUGUST ISSUE we published the proceedings of the American Dental Association at Cincinnati, Aug. 1—4, '82. This report was written by one of the most able members of that body, expressly for the INDEPENDENT PRACTITIONER. His modesty required that we withhold his name.

The first article in said issue, entitled "Homœopathy," slipped in through the printers, into whose hands it fell during our absence, and we did not read it until after it had been mailed. We truly regret that it has wounded some of our chaste readers.

